# Expression profiling and functional characterization of miR-192 throughout sheep skeletal muscle development

**DOI:** 10.1038/srep30281

**Published:** 2016-07-25

**Authors:** Qian Zhao, Ye Kang, Hong-Yang Wang, Wei-Jun Guan, Xiang-Chen Li, Lin Jiang, Xiao-Hong He, Ya-Bin Pu, Jian-Lin Han, Yue-Hui Ma, Qian-Jun Zhao

**Affiliations:** 1 Institute of Animal Science, Chinese Academy of Agricultural Sciences, Beijing 100193, China; 2 CAAS-ILRI Joint Laboratory on Livestock and Forage Genetic Resources, Institute of Animal Science, Chinese Academy of Agricultural Sciences, Beijing 100193, China

## Abstract

MicroRNAs (miRNAs) are evolutionarily conserved, small, non-coding RNAs that have emerged as key regulators of myogenesis. Here, we examined the miRNA expression profiles of developing sheep skeletal muscle using a deep sequencing approach. We detected 2,396 miRNAs in the sheep skeletal muscle tissues. Of these, miR-192 was found to be up-regulated in prenatal skeletal muscle, but was down-regulated postnatally. MiR-192 expression also decreased during the myogenic differentiation of sheep satellite cells (SCs). MiR-192 overexpression significantly attenuated SCs myogenic differentiation but promoted SCs proliferation, whereas miR-192 inhibition enhanced SCs differentiation but suppressed SCs proliferation. We found that miR-192 targeted retinoblastoma 1 (*RB1*), a known regulator of myogenesis. Furthermore, knockdown of *RB1* in cultured cells significantly inhibited SCs myogenic differentiation but accelerated SCs proliferation, confirming the role of *RB1* in myogenesis. Taken together, our findings enrich the ovine miRNA database, and outline the miRNA transcriptome of sheep during skeletal muscle development. Moreover, we show that miR-192 affects SCs proliferation and myogenic differentiation via down-regulation of *RB1*.

Sheep are important farm animals worldwide, producing high quality meat with the ability to adapt to different climatic and grazing conditions. An improved understanding of the molecular mechanisms of muscle development (myogenesis) in ovine may increase meat productivity in the sheep and lamb industry. In adults, the cells responsible for producing myoblasts in skeletal muscle are satellite cells (SCs), which are located beneath the basal lamina of the myofibre. After skeletal muscle suffers injury, SCs fuse together to allow new myotube formation, or fuse to injured myofibres to replace lost myonuclei^1^. As myonuclei are post-mitotic, SCs serve as an excellent model to study the molecular mechanisms underlying muscle development. Previous studies show that in order for skeletal muscle regeneration to take place, SCs must express Pax7, a transcription factor that regulates cell proliferation^2^,^3^,^4^,^5^. Moreover, it was recently suggested that microRNA-1 (miR-1) and miR-206 regulate the SCs proliferation and myogenic differentiation by repressing Pax7^6^. Therefore, microRNAs (miRNAs) appear to be important for SCs proliferation and differentiation.

MiRNAs are short non-coding RNAs that modulate gene expression through translational repression and mRNA decay^7^, and have recently been profiled in a variety of tissues from different animals^8^,^9^,^10^. However, few studies have focused on the identification and profiling of miRNAs in sheep skeletal muscle. Such profiling of miRNAs in skeletal muscle could enhance our understanding of the effect of miRNAs on myogenesis. Indeed, recent studies have identified several miRNAs involved in myogenesis based on miRNAs expression profiling of skeletal muscle. For example, in mouse, miR-1 and miR-206 are now considered as muscle-specific miRNAs, which promote muscle differentiation by targeting histone deacetylase 4 (HDAC4) and DNA polymerase α (Pola1), respectively^11^,^12^. These miRNAs are also implicated in sheep myogenesis. For example, in Texel sheep, a G to A transition in the three prime untranslated region (3′-UTR) of myostatin (GDF8) creates a target site for miR-1 and miR-206, and in turn, these miRNAs inhibit the expression of GDF8 resulting in muscular hypertrophy^13^. In mouse, skeletal myogenesis is also regulated by several other ubiquitously expressed miRNAs, including: miR-125b^14^, miR-26a^15^, miR-27^16^, miR-29^17^, miR-486^18^, miR-378^19^, and miR-155^20^.

In sheep, miR-192 was previously found to be expressed in skeletal muscle^21^, although its precise function remains unclear. Several studies indicate that miR-192, which was first cloned from mouse and human^22^, is involved in disease pathogeneses, including atrial fibrillation, aristolochic acid nephropathy, and diabetic kidney glomeruli^23^,^24^,^25^. In humans, miR-192 is also known as a tumor-related miRNA: up-regulation of miR-192 represses breast cancer cell proliferation^26^, and inhibits lung cancer cell proliferation, as well as inducing cell apoptosis^27^. Moreover, in colon cancer, miR-192 regulates dihydrofolate reductase and cellular proliferation through the p53 tumor suppressor network^28^. However, little is known about the function of miR-192 in skeletal myogenesis.

In addition to the above miRNAs, retinoblastoma 1 (*RB1*) is also implicated in skeletal myogenesis^29^. Retinoblastoma 1 protein (pRb) belongs to a family of three proteins, which also includes retinoblastoma-like 1 (RBL1/p107) and retinoblastoma-like 2 (RBL2/p130)^30^. pRb critically regulates cell cycle progression by interacting with the E2F transcription factor family of proteins^31^,^32^; however, it can also control cell cycle progression via E2F-independent ways^33^. *RB1* is also known as a tumor suppressor gene, as it is functionally inactivated in most human cancers^34^. However, increasing evidence suggests that pRb is also a key regulator of murine muscle development^29^. pRb is a tumor suppressor protein that also restricts the cell’s ability to replicate DNA, so inhibition of this protein will decrease myogenesis.

Here, we examined miRNA expression during ovine skeletal muscle development by deep sequencing of skeletal muscle obtained from local Chinese Duolang sheep that is bred for meat and fat. We investigated the significantly differentially expressed miRNAs at each distinct stage of muscle development (from fetal to 3 years postnatal). We then further explored the functional mechanisms of one differentially expressed miRNA (miR-192) that has previously been implicated in myogenesis, and found that it targeted *RB1*. The overall aim of this study was to improve our understanding of miRNA function during muscle development in ovine.

## Results

### Differential expression profile of microRNAs from developing sheep skeletal muscle

To explore the potential function of miRNAs in sheep skeletal myogenesis, we examined the expression of miRNAs using deep sequencing at four distinct stages of muscle development: fetus 90 days (F90), fetus 110 days (F110), lamb 40 days (L40) and adult 3 years (A3Y). A total of 15,711,484, 14,075,573, 15,452,905 and 15,832,379 reads were obtained for the F90, F110, L40 and A3Y libraries, respectively. The analysis of length distribution showed that the majority of the reads had lengths of 21–23 nt (Figure S1). Among these reads, 14,746,030 at F90, 13,223,764 at F110, 11,043,952 at L40, and 11,290,187 at A3Y were mappable to the *Ovis aries* genome. In total, 2,396 miRNAs were identified in the four small RNA libraries. Of the total miRNAs, 80.1% (1920/2396 miRNAs) were predicted to be new miRNAs that were not deposited in the miRBase database. Of the predicted new miRNAs, 37.0% (711/1920 miRNAs) were conserved in other species (cow, human, mouse, etc.), and 63.0% (1209/1920 miRNAs) were unannotated miRNAs (Table S1). The most abundant known sheep miRNAs in ovine skeletal muscle are listed in Table 1.

**Figure 1:**
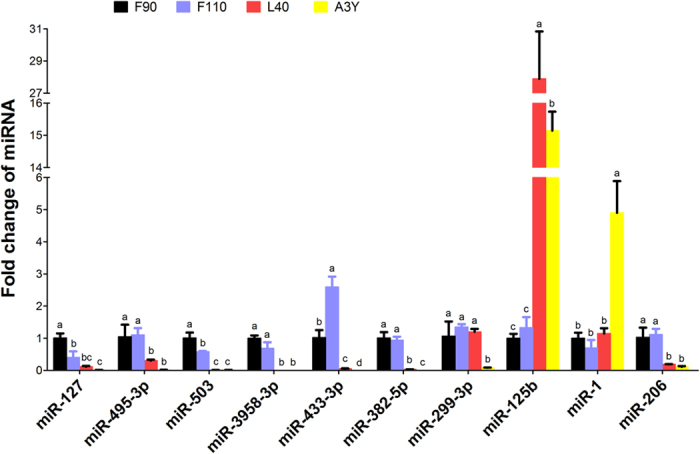
Validation of 10 differentially expressed miRNAs found in sheep skeletal muscle at four developmental stages by qPCR. The results are shown as the mean ± SD of three replicates. One-way ANOVA followed by Duncan’s test was performed to determine statistical significance. Superscript letters (a–d) indicate significant differences (*P* < 0.05).

Next, we compared the miRNA expression profiles at the four developmental stages (F90, F110, L40, and A3Y). In total, 15 significantly differently expressed miRNAs (SDEmiRs) were identified between F90 *vs.* F110 (Table S2), 290 SDEmiRs were identified between F90 *vs.* L40 (Table S3), 343 SDEmiRs were identified between F90 *vs.* A3Y (Table S4), 207 SDEmiRs were identified between F110 *vs.* L40 (Table S5), 209 SDEmiRs were identified between F110 *vs.* A3Y (Table S6) and 293 SDEmiRs were identified between L40 *vs.* A3Y (Table S7). Furthermore, to validate the miRNA deep sequencing data (according to Table S1), quantitative real-time PCR (qPCR) was performed to verify 10 differently expressed miRNAs, including: miR-127, miR-495-3p, miR-503, miR-3958-3p, miR-433-3p, miR-382-5p, miR-299-3p, miR-125b, miR-1, and miR-206. The relative expression of these miRNAs was highly correlated with the sequence data (Fig. 1).

### MiRNA target prediction, gene ontology enrichment, and Kyoto Encyclopedia of Genes and Genomes (KEGG) pathway analysis of target genes

The TargetScan program was applied to predict the targets of the SDEmiRs (Table S8,S9,S10,S11,S12–S13). Gene ontology term and KEGG pathway enrichment analyzes were conducted for the targets of SDEmiR. The targets of SDEmiRs were grouped into three gene ontology categories: biological process, cellular component, and molecular function (Figure S2). Furthermore, KEGG pathway analyzes identified some significantly enriched pathways, such as focal adhesion, glioma, PI3K-AKT signaling pathway and oxidative phosphorylation (Figure S3).

### MiR-192 may be involved in sheep skeletal muscle development

**Figure 2:**
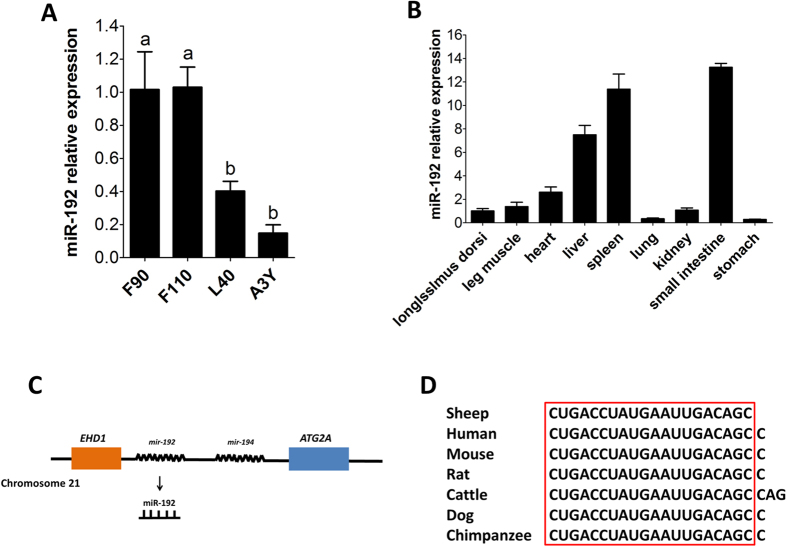
MiR-192 expression during muscle growth in the developing sheep. (**A**) Relative expression of miR-192 at F90, F110, L40, and A3Y as detected by qPCR. (**B**) Tissue distribution of miR-192 examined by qPCR in adult sheep. The fold change of miR-192 was relative to miR-192 expression of *longissimus dorsi*. (**C**) Schematic representation of the genomic location of miR-192. (**D**) The conservation of mature miR-192 from seven different species. The results are shown as the mean ± SD of three replicates. In A, one-way ANOVA followed by Duncan’s test was performed to determine statistical significance. Superscript letters (a, b) indicate significant differences (*P* < 0.05).

According to our miRNA profiling in sheep, we found miR-192 was down-regulated by 5-fold in postnatal skeletal muscle of A3Y compared with prenatal skeletal muscle of F90. The expression of miR-192 in developing sheep skeletal muscle was confirmed by qPCR (Fig. 2A). Tissue expression profiling of miR-192 showed that miR-192 was ubiquitously expressed in different tissues, including skeletal muscle (*semitendinosus* and *longissimus dorsi*) (Fig. 2B). Moreover, miR-192 was up-regulated in skeletal muscle from Lamin A/C (LMNA)-mutated patients with abnormal muscle development^35^, indicating that it was closely related to myogenesis. Ovine *mir-192* is an intergenic miRNA gene, which is adjacent to *mir-194* and located between *EHD1* and *ATG2A* at chromosome 21 (Fig. 2C). The 20 nucleotides comprising the sheep mature miR-192 are completely conserved across species, although the sheep miR-192 lacks an additional “C”, and the cattle miR-192 has an extra “AG” (Fig. 2D). Altogether, these results indicate that miR-192 decreases during muscle growth in the developing sheep and is an attractive candidate that may be involved in myogenesis.

### MiR-192 is down-regulated during myogenic differentiation of sheep SCs and murine C2C12 myoblasts

**Figure 3:**
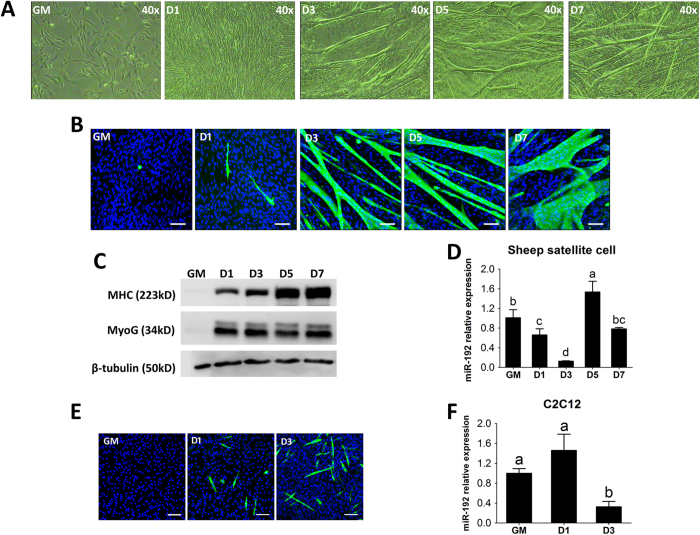
MiR-192 expression during myogenic differentiation of sheep satellite cells (SCs) and murine C2C12 myoblasts. (**A**) Microscopic images of sheep skeletal muscle SCs cultured in growth medium (GM) or differentiation medium for 1, 3, 5, and 7 days (D1, D3, D5, and D7). (**B**) The undifferentiated (GM) or differentiated SCs on various days (D1, D3, D5, and D7) were fixed and immunostained for MHC (green) and DAPI (blue). Bars, 100 μm. (**C**) Immunoblotting for detecting MHC and MyoG protein expression in cells cultured as described in A. β-tubulin was used as the reference gene. (**D**) MiR-192 expression was determined by qPCR in undifferentiated (GM) or differentiated SCs on various days (D1, D3, D5, and D7). (**E**) Murine C2C12 myoblasts cultured in GM or at day 1 (D1) or day 3 (D3) after differentiation were fixed and immunostained for MHC (green) and DAPI (blue). Bars, 100 μm. (**F**) MiR-192 expression was detected by qPCR in C2C12 myoblasts cultured as described in E. In A, B, C and E, representative results of three replicates are shown. In D and F, results are shown as the mean ± SD of three replicates. One-way ANOVA followed by Duncan’s test was performed to determine statistical significance. Superscript letters (a-d) indicate significant differences (*P* < 0.05).

To further research the possible role of miR-192, we separated sheep skeletal muscle SCs. We collected leg muscle samples of ovine fetus and then carefully separated SCs according to a previous study^36^. The expression of Pax7 in the SCs was detected by immunofluorescence, and the SCs were consistently >90% pure (Figure S4). The SCs formed significant myotubes by day 3 after they were induced to differentiate (Fig. 3A,B). Moreover, myogenin (MyoG), an early myogenic marker, and myosin heavy chain (MHC), a late myogenic marker, were both normally expressed during SCs differentiation (Fig. 3C). To explore the expression of miR-192 during SCs differentiation, we examined miR-192 levels during SCs differentiation. The results showed that the miR-192 levels in SCs declined on days 1 and 3 of differentiation (Fig. 3D), which was a period of myotube formation (Fig. 3A–C). However, as miR-192 expression increased again on days 5 and 7 during the myogenic differentiation of sheep SCs (Fig. 3D), additional mechanisms may control miR-192 levels. Similarly, miR-192 was also down-regulated in C2C12 myoblasts on day 3 of differentiation (Fig. 3E,F). These results indicate that miR-192 is down-regulated during SCs and C2C12 myoblasts differentiation.

### MiR-192 negatively regulates myogenic differentiation of sheep SCs and murine C2C12 myoblasts

**Figure 4:**
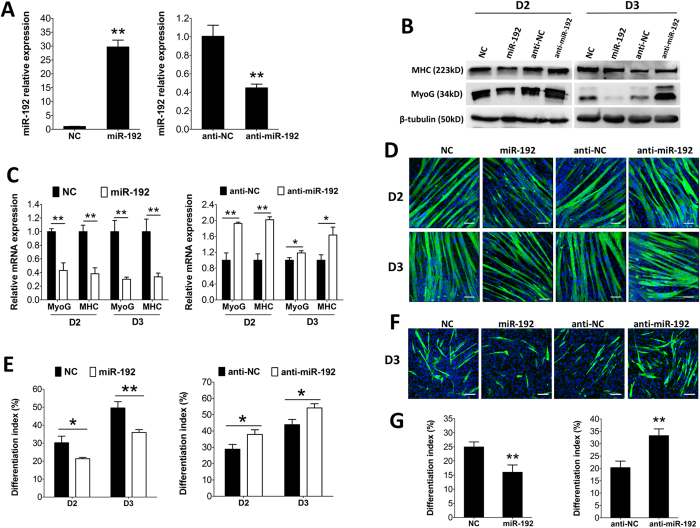
MiR-192 regulation of myogenic differentiation in sheep SCs and murine C2C12 myoblasts. (**A**) MiR-192 expression after transfection with miR-192 mimics (miR-192), negative control (NC), 2′-O-methyl antisense oligonucleotides against miR-192 (anti-miR-192) and single-stranded negative control (anti-NC) determined by qPCR 48 h after transfection (n = 3). (**B**) Representative results of immunoblotting for the protein expression of MHC and MyoG at D2 and D3 after transfection (n = 3). (**C**) MHC and MyoG expression determined by qPCR at D2 and D3 after transfection (n = 3). (**D**) Representative results of immunofluorescence analysis of MHC (green) and DAPI (blue) at D2 and D3 after transfection (n = 5). Bars, 100 μm. (**E**) Quantification of the differentiation index based on D (n = 5). (**F**) Representative results of immunofluorescence analysis of MHC (green) and DAPI (blue) at D3 after transfection in C2C12 myoblasts (n = 5). Bars, 100 μm. (**G**) The differentiation index was quantified in F (n = 5). In A, C, E and G, results are shown as the mean ± SD. *P < 0.05; **P < 0.01. Unpaired two-tailed *t*-test was performed to calculate *p*-value.

To examine the function of miR-192 in myogenic differentiation, we introduced miR-192 mimics and negative control (NC) into SCs. The SCs were transfected with miR-192 mimics or NC in growth medium (GM) (Fig. 4A). Then the cells were induced to differentiate in differentiation medium (DM). The introduction of miR-192 decreased the protein levels of MyoG and MHC by days 2 and 3 of differentiation (Fig. 4B). Similarly, a significant decrease in the mRNA expression levels of MyoG and MHC was observed on days 2 and 3 of differentiation (Fig. 4C). Moreover, overexpression of miR-192 dampened myotube formation (Fig. 4D), as confirmed by a decreased differentiation index (Fig. 4E).

We also introduced the 2′-O-methyl antisense oligonucleotides against miR-192 (anti-miR-192) and single-stranded anti-NC into SCs (Fig. 4A). Inhibition of miR-192 enhanced the protein and mRNA expression of myogenic markers (Fig. 4B,C), and this was accompanied by enhanced myotubes formation (Fig. 4D,E). Next, we explored the role of miR-192 in murine C2C12 myoblasts differentiation. Similarly, overexpression of miR-192 inhibited C2C12 myoblasts differentiation, while inhibition of miR-192 promoted differentiation (Fig. 4F,G). Taken together, our results indicate that miR-192 plays a negative role in the myogenic differentiation of SCs and C2C12 myoblasts.

### MiR-192 accelerates proliferation of sheep SCs

**Figure 5:**
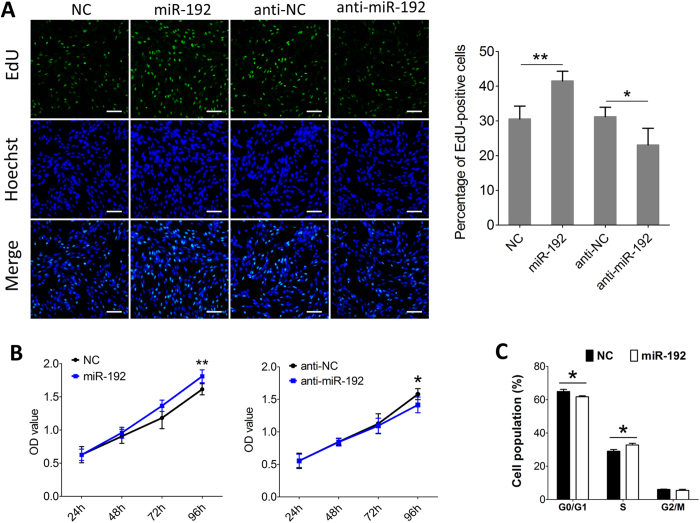
MiR-192 accelerates proliferation of sheep SCs. (**A**) Representative photoimages of EdU assay of SCs after transfection (left) and quantification of EdU-positive cells (right) (n = 5). Bars, 100 μm. (**B**) MTT assay of SCs at 24 h, 48 h, 72 h and 96 h after transfection (n = 5). (**C**) Cell cycle analysis using flow cytometry of SCs after transfection with miR-192 or NC (n = 3). The results are shown as the mean ± SD. *P < 0.05; **P < 0.01. Unpaired two-tailed *t*-test was performed to calculate *p*-value.

Next, we examined the role of miR-192 on proliferation of sheep SCs using 5-Ethynyl-2′-deoxyuridine (EdU) cell proliferation assay. We found that overexpression and inhibition of miR-192 increased and decreased the proportion of EdU-positive cells (Fig. 5A), respectively, indicating that miR-192 promoted proliferation of SCs. Moreover, the MTT assay further confirmed that miR-192 enhanced SCs proliferation (Fig. 5B). Furthermore, we analyzed the cell cycle by flow cytometry after transfection, and found that the introduction of miR-192 decreased the G0/G1 population of cells but increased the S population of cells, suggesting that miR-192 overexpression inhibited cell cycle arrest (Figs 5C and S5). Taken together, these results suggest that miR-192 accelerates SCs proliferation.

### MiR-192 directly targets 3′-UTR of *RB1*

**Figure 6:**
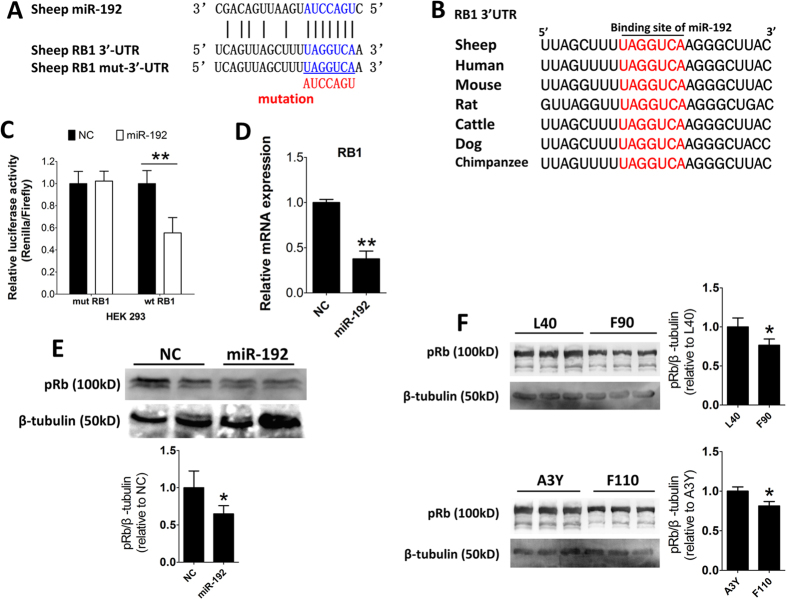
MiR-192 directly targets *RB1*. (**A**) The predicted binding site (blue) and mutated site (red) of miR-192 in the 3′-UTR of sheep *RB1* are shown. (**B**) The conservation of the miR-192 binding site in the 3′-UTR of *RB1* from seven different species. (**C**) Dual-luciferase activity assay of the wild-type (wt) or mutant 3′-UTR (mut) of *RB1*. MiR-192 mimics (miR-192) or NC were co-transfected with the wild-type or mutant 3′-UTR luciferase reporters of *RB1* in HEK293 cells (n = 3). (**D**) Expression of *RB1* mRNA in SCs following transfection with miR-192 mimics (miR-192) or NC examined by qPCR. Total RNAs were harvested at 48 h after transfection (n = 3). (**E**) Representative results of immunoblotting of SCs after transfection. Total proteins were harvested at 48 h after transfection. Quantification of pRb was normalized to β-tubulin (n = 4). (**F**) Immunoblotting for the pRb expression of ovine skeletal muscle tissue at F90, F110, L40, and A3Y. Quantification of pRb was normalized to β-tubulin. The results are shown as the mean ± SD. *P < 0.05; **P < 0.01. In C, D and F, unpaired two-tailed *t*-test was performed to calculate *p*-value. In E, paired two-tailed *t*-test was performed to calculate *p*-value.

pRb plays a critical role in the differentiation of myoblasts; pRb-deficient myoblasts lose the ability to form multinucleated myotubes^29^. Using TargetScan and PicTar, we found that the sheep *RB1* 3′-UTR has a highly conserved binding site for miR-192 (Fig. 6A,B). To examine whether miR-192 targets the *RB1* 3′-UTR, we constructed luciferase reporters that included a fragment of either the wild-type or mutant *RB1* 3′-UTR (Fig. 6A). The miR-192 mimics or NC were co-transfected with the reporters into human HEK293 cells, a model cell line which has stable efficiency of transfection. MiR-192 significantly reduced the activity of wild-type reporter of *RB1* 3′-UTR; however, no reduction in activity was observed with the mutant reporter of *RB1* 3′-UTR (Fig. 5C). This confirms that miR-192 directly targets the 3′-UTR of *RB1*. Furthermore, the introduction of miR-192 repressed the *RB1* mRNA and protein expression in SCs (Fig. 6D,E). Moreover, the expression level of pRb was significantly higher in L40 than in F90, as well as higher in A3Y than in F110 (Fig. 6F). These results showed that the expression of pRb has a negative correlation with miR-192 expression in developing animal (Figs 2A and 6F), further confirming that *RB1* is a target gene of miR-192. Together, these results suggest that the protein and mRNA expression of *RB1* are directly inhibited by miR-192.

### Knockdown of *RB1* inhibits myogenic differentiation but promotes proliferation of cultured sheep SCs

**Figure 7:**
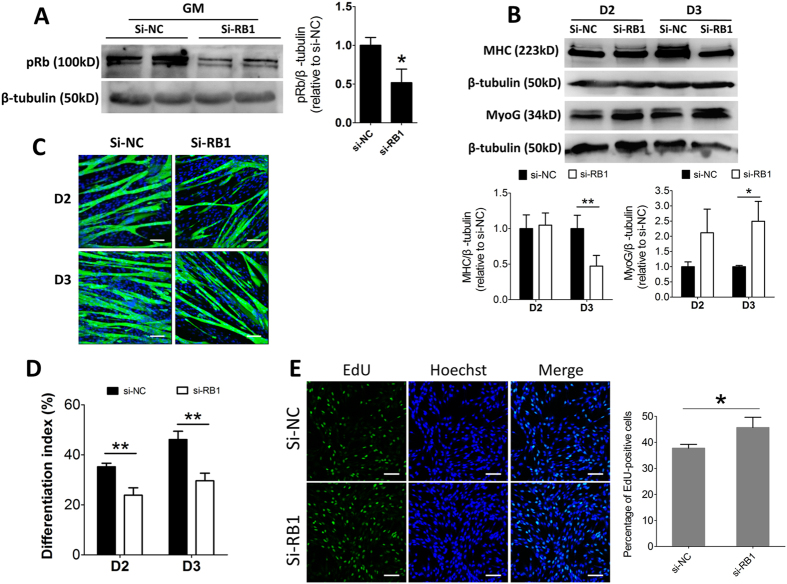
Knockdown of *RB1* inhibits myogenic differentiation but promotes proliferation of cultured SCs. (**A**) Representative results of immunoblotting for the pRb at 48 h after transfection with siRNA against sheep *RB1* (si-RB1) or siRNA control (si-NC). Quantification of pRb was normalized to β-tubulin (n = 4). (**B**) Representative results of immunoblotting analysis of MHC and MyoG at D2 and D3 after transfection with si-RB1 or si-NC. Quantification of MHC and MyoG was normalized to β-tubulin (n = 3). (**C**) Representative results of immunofluorescence analysis of MHC (green) and DAPI (blue) at D2 and D3 after transfection with si-RB1 or si-NC (n = 3). Bars, 100 μm. (**D**) Quantification of the differentiation index based on C (n = 3). (**E**) Representative photoimages of EdU assay of SCs after transfection with si-*RB1* or si-NC and quantification of EdU-positive cells (n = 5). Bars, 100 μm. The results are shown as the mean ± SD. *P < 0.05; **P < 0.01. In A and B, paired two-tailed *t*-test was performed to calculate *p*-value. In D and E, unpaired two-tailed *t*-test was performed to calculate *p*-value.

To examine the role of pRb on myogenic differentiation and proliferation of sheep SCs, we silenced endogenous *RB1* of SCs by siRNA. SiRNA against sheep *RB1* (si-*RB1*) significantly diminished its protein expression (Fig. 7A). Moreover, the reduction of pRb significantly repressed SCs differentiation, as indicated by the inhibition of MHC protein expression and formation of myotubes (Fig. 7B–D). The fact that miR-192 overexpression resulted in down-regulation of MyoG protein expression (Fig. 4B), but that si-*RB1* increased MyoG protein expression (Fig. 7B), implies that other target genes of miR-192 may be involved in MyoG expression. Indeed, the increased MyoG expression caused by si-*RB1* was consistent with a previous study reporting pRb-deficient myoblasts up-regulated MyoG expression^29^. Moreover, si-*RB1* accelerated SCs proliferation (Fig. 7E), which was very similar to the impact of the overexpression of miR-192. Thus, *RB1* knockdown significantly represses SCs myogenic differentiation, but accelerates SCs proliferation, confirming the role of pRb in myogenesis.

## Discussion

MiRNA profiling analysis based on deep sequencing is a powerful tool for functional studies of miRNA. In this study, 2,396 miRNAs, including 1,920 novel miRNAs, were identified from four developmental stages of sheep skeletal muscles. Among these miRNAs identified, we found some that were differentially expressed, including miR-192. Further study demonstrated that miR-192 was down-regulated during the myogenic differentiation of sheep SCs and murine C2C12 myoblasts. We found that miR-192 repressed myogenic differentiation but accelerated proliferation of sheep SCs. Dual-luciferase reporter assay confirmed that *RB1* was a direct target of miR-192. Moreover, knockdown of endogenous *RB1* by siRNA promoted proliferation but inhibited myogenic differentiation of sheep SCs, confirming the role of *RB1* in myogenesis.

MiR-192 is an attractive candidate for regulating skeletal muscle development. It was previously thought to be a regulator of p53, a human tumor suppressor, and is overexpressed in gastric cancer^28^,^37^. Moreover, miR-192 has been identified as a potential therapeutic target or biomarker for drug-induced liver damage^38^. MiR-192 targets various genes in different cells and organs^24^,^25^,^26^,^27^,^28^, indicating that it is extensively involved in biological processes. Indeed, this is consistent with our tissue expression profile showing that miR-192 was ubiquitously expressed in different sheep tissues. However, whether miR-192 regulates myogenesis has not been clarified.

A previous study demonstrated that miR-192 expression was higher in the muscle of Small Tail Han sheep, a Chinese breed with a slower growth rate, than in muscle from Dorset sheep that yield a large and lean carcass^21^. Studies have revealed that fetus 80–120 days is the vital stage for ovine myofibre formation and proliferation^39^,^40^. In our study, we found that miR-192 expression was significantly higher in prenatal skeletal muscle of fetus 90 days, a period which would expect a significant amount of muscle progenitor cell proliferation occurring, than in postnatal muscle of adult 3 years, a period which myofibres were well established. These facts indicated that miR-192 could be involved in skeletal muscle development.

We then confirmed that miR-192 regulated proliferation and differentiation of SCs by targeting *RB1*. *RB1* is not only implicated in childhood retinoblastoma, bladder cancer, and lung cancer, but acts as a negative regulator of the cell cycle and is involved in cell senescence, growth arrest, apoptosis, and differentiation^30^. Recently, several miRNAs were found to participate in the regulation of cancer cell proliferation via targeting *RB1*. In gastric cancer cells, miR-215 was able to target *RB1* to modulate gastric cancer cell proliferation^41^, and miR-132 also promoted gastric cancer cell proliferation via targeting *RB1*^42^. In meningioma cells, miR-335 enhanced cell proliferation through targeting *RB1*^43^. Furthermore, a number of studies indicate *RB1* is critical for muscle development. For example, myoblasts expressing low levels of *RB1* fail to develop normally and accumulate large polyploid nuclei^44^, and mice deficient for the *RB1* gene die at mid-gestation^45^,^46^. Moreover, *RB1* was previously shown to be required during myogenic differentiation, and modulate the progression of differentiation^29^. Indeed, a lack of *RB1* accelerates cell cycle re-entry of quiescent SCs and increases the number of SCs; however, SCs terminal differentiation was greatly diminished^47^. Similarly, we demonstrated here that miR-192 attenuated sheep SCs differentiation but accelerated proliferation, which was characterized by directly repressing the expression of *RB1*.

Another study found that miR-192 was markedly up-regulated in skeletal muscle from Lamin A/C (LMNA)-mutated patients with skeletal muscle dystrophy^35^. In addition, LMNA null muscle shows delayed expression of *RB1*, suggesting that the dominant LMNA mutations may alter *RB1* function^48^. However, the mechanism of delayed expression of *RB1* in LMNA null muscle is elusive. Here, we demonstrated that miR-192 negatively regulates myogenic differentiation through repressing the expression of *RB1*. Thus, we speculate that in LMNA-related muscular dystrophy, the up-regulation of miR-192 delays the expression of *RB1* by directly targeting its 3′-UTR, and in turn, this may lead to abnormalities in skeletal muscle development.

In conclusion, the results of our study indicate that miR-192 is important for myogenic differentiation and proliferation, and its up-regulation can negatively affect myogenesis. Myogenic differentiation was delayed by the introduction of miR-192, and promoted by miR-192 inhibition. We also found that miR-192 directly targets the *RB1* gene at the post-transcriptional level, and that *RB1* knockdown significantly represses SCs myogenic differentiation but accelerates SCs proliferation. These results reveal a miRNA-mediated regulation mechanism, by which miR-192 regulates myogenic differentiation and proliferation of skeletal muscle SCs through the repression of *RB1*.

## Methods

### Animals and tissue samples collection

All animal experimental procedures (i.e., on Duolang sheep from Sinkiang, China) were performed according to the guidelines for the care and use of experimental animals established by the Ministry of Agriculture of People’s Republic of China. All experimental protocols were approved by Science Research Department of the Institute of Animal Sciences, Chinese Academy of Agricultural Sciences (CAAS) (Beijing, China). The fetal longissimus dorsi were collected from three fetuses of Duolang sheep at 90 days post coitus, and from another three fetuses at 110 days post coitus. The lamb longissimus dorsi were collected from three male Duolang lambs at 40 days postnatal. The adult longissimus dorsi were collected from three male Duolang sheep at 3 years postnatal. Nine different tissues (including semitendinosus, longissimus dorsi, small intestine, liver, spleen, lung, heart, kidney, and stomach) were collected from the three male Duolang sheep at 40 days postnatal. All animals were euthanized. All tissues were immediately frozen in liquid nitrogen after harvesting.

### Library construction and miRNA deep sequencing

RNAs were extracted with TRIzol (Invitrogen, USA) from the *longissimus dorsi* of Duolang sheep fetuses at 90 days (F90) and 110 days (F110), lambs at 40 days (L40), and adults at 3 years (A3Y), according to the manufacturer’s protocols. The quality (RIN ≥ 8 and 28S/18S ≥ 1.0) and quantity of the RNA samples were detected on a Bioanalyzer 2100 system using an RNA 6000 Nano kit (Agilent Technologies, USA). The RNA from the three Duolang sheep in each group were pooled to generate the four libraries (F90, F110, L40, and A3Y), which were prepared according to Illumina’s instructions (Illumina GAIIx, Illumina, USA), and sequenced by Shanghai Biotechnology Corporation.

### MiRNA profiling and novel miRNA prediction

Clean reads were obtained by removing low quality reads, reads with 5′ adaptor pollution or poly (A) stretches, reads <18 nt, and reads without 3′ adaptors. The clean reads were mapped to the *Ovis aries* genome (version 4.0) using the SOAP program (http://soap.genomics.org.cn/). After mapping, the reads were blasted against the Rfam database (http://www.sanger.ac.uk/software/Rfam) and the GenBank non-coding RNA database (http://blast.ncbi.nlm.nih.gov/). After removal of mRNA, snRNA, tRNA, rRNA, and snoRNA, the remaining reads were then searched against the mature miRNAs of chimpanzee, human, cow, horse, mouse, rhesus monkey, western lowland gorilla, bonobo, Tasmanian devil, platypus, pig, gray short-tailed opossum and sheep in miRBase (release 20) to identify known conserved miRNA homologs in the sheep. Only the mature and precursor sequences of small RNAs that perfectly matched to known ovine miRNAs in the miRBase were identified as known ovine miRNAs. To predict potential new miRNAs, the 150 bases upstream and downstream flanking the remaining reads were obtained, and secondary structures were detected using the miRDeep program. If a perfect stem-loop structure was formed, the miRNA sequence was located at one arm of the stem, the miRNA sequence ranged between 18–26 nt, and its free energy of hybridization was lower than −20 kcal/mol, then the miRNA was considered a potential novel miRNA. The potential novel miRNAs with predicted hairpin structures were mapped to conserved miRNAs of other species (chimpanzee, human, cow, horse, mouse, rhesus monkey, western lowland gorilla, bonobo, tasmanian devil, platypus, gray short-tailed opossum and pig) in the miRBase. The remaining unmapped but hairpin-structured reads were considered as potential new miRNAs without known miRNA annotations.

DEGseq software was applied to identify SDEmiRs and a MA-plot-based random sampling model (MARS) was performed to calculate the *p*-value. If |log2 (ratio miRNA levels)| > 1 and *p* < 0.05, the miRNAs were identified as significantly differentially expressed between the two developmental stages of skeletal muscle.

### MiRNA targets prediction, gene ontology enrichment and KEGG pathway analyzes

The TargetScan program (http://www.targetscan.org) was employed to predict targets of all the SDEmiRs. The target genes of SDEmiRs were analyzed with gene ontology and association with different pathways was conducted with KEGG (http://www.genome.jp/kegg), Biocarta (www.biocarta.com), and Reatome (http://www.reactome.org/) databases.

### Cell culture

Human HEK293 and murine C2C12 myoblast cells were purchased from the China Infrastructure of Cell Line Resource. C2C12 myoblasts were cultured in growth medium (GM) consisting of Dulbecco’s modified Eagle’s medium (DMEM) (Gibco, USA) supplemented with 10% FBS (Gibco, USA) at 37 °C with 5% CO_2_. Myogenic differentiation was induced by a differentiation medium (DM) with DMEM supplemented with 2% horse serum (Gibco, USA). HEK293 cells were maintained in DMEM with 10% FBS at 37 °C with 5% CO_2_.

The sheep skeletal muscle SCs were enzymatically isolated from leg muscle tissues obtained from five Duolang sheep fetuses at 90 days, using a two-step digestion method, as described previously^36^. Briefly, leg muscle tissues were excised, cut into small pieces, digested with 0.1% type I collagenase (Sigma-Aldrich, USA) for 1 h, and then digested with 0.25% trypsin (Gibco, USA) for 20 min. The samples were vortexed every 10 min and filtered through a 200-mesh sieve, and SCs were purified using the differential adhesion method^49^. Sheep skeletal muscle SCs were proliferated in GM consisting of DMEM/F-12 (Gibco, USA) supplemented with 20% FBS and 10% horse serum in a collagen type 1-coated plate, and then differentiated using DMEM/F-12 with 2% horse serum.

### RNA isolation, RT-PCR and quantitative real-time PCR

Total RNA was extracted from the *longissimus dorsi* muscles, C2C12 myoblasts, or sheep skeletal muscle SCs with TRIzol reagent (Invitrogen, USA), according to manufacturer’s instructions. For mRNA quantification, 1 μL of total RNA (1000 ng/μL) was reverse transcribed into cDNA using the PrimeScript RT reagent Kit (Perfect Real Time; Takara, Japan) for quantitative real-time PCR (qPCR). *GAPDH* and *ACTB* genes were used as internal normalization controls. qPCR was carried out in an Applied Biosystems 7500 Real-Time Detection system using the SYBR premix Ex Taq qPCR Kit (Takara, Japan).

For miRNA quantification, a miRNA stem-loop primer and a pair of primers were designed for miRNA reverse transcription and qPCR, respectively. The *U6* gene was used as the internal normalization control. MiRNA stem-loop was reverse transcribed with the PrimeScript RT reagent Kit and quantified by qPCR using the SYBR premix Ex Taq qPCR Kit, according to manufacturer’s protocols. Relative gene expression was determined by the 2^−ΔΔCt^ method^50^,^51^. The primers used for qPCR are listed in Supplementary Tables S14 and S15.

### Immunoblotting and immunofluorescence

All protein preparations were quantified using the BCA Protein Assay Kit (Thermo Scientific, USA). Immunoblotting was performed using standard procedures and antibodies against pRb (1:1000, Abcam, UK), MHC (1:200, Developmental Studies Hybridoma Bank, USA), MyoG (1:100, Developmental Studies Hybridoma Bank, USA), β-tubulin (1:2000, Sigma-Aldrich, USA), and GAPDH (1:5000, Sigma-Aldrich, USA). Protein abundance was analyzed by ImageJ tool. Quantification of protein was normalized to β-tubulin.

For immunofluorescence, SCs and C2C12 myoblasts were plated in 6-well plates and induced to differentiate. The cells were harvested, fixed with 4% paraformaldehyde for 20 min, treated with 0.25% Triton X-100 for 15 min, and then blocked with goat serum (Bioss, China) for 1 h. After blocking, the cells were incubated with primary antibody against murine and ovine MHC (1:100, Developmental Studies Hybridoma Bank, USA) or Pax7 (1:200, Bioss, China) overnight at 4 °C. The AlexaFluor-488 conjugated mouse IgG (1:1000; Invitrogen, USA) was incubated for 1 h at room temperature. The cell nuclei were stained with DAPI (Sigma-Aldrich, USA). The differentiation index (percentage of nuclei in MHC) was calculated as previously described^14^.

### Cell proliferation assay

Three separate assays were applied to quantify the proliferation of sheep skeletal muscle SCs. First, flow cytometry was used to analyze the phase distribution of the cell cycle. The cultured sheep skeletal muscle SCs in GM were collected 48 h after transfection and fixed in 75% ethanol overnight at −20 °C. Then the cells were incubated with 50 μg/ml propidium iodide (Sigma-Aldrich, USA) containing 10 mg/ml RNase A (Takara, Japan) and 0.2% (v/v) Triton X-100 (Sigma-Aldrich, USA) for 30 min at 4 °C. The cell cycle distribution was analyzed using a CytomicsTM FC 500 flow cytometer (Beckman Coulter, USA). Second, the 5-Ethynyl-2′-deoxyuridine (EdU) (Guangzhou RiboBio, China) cell proliferation assay was used to measure SCs proliferation, according to manufacturer’s protocols. Briefly, after transfection in GM, the sheep skeletal muscle SCs were seeded in 96-well plate and cultured in GM for 24 h. Then, the cells were incubated 2 h with medium containing 50 μM EdU before immunostaining. Images were collected using a fluorescence microscope (Nikon, Japan). The ratio of EdU-positive cells was calculated with (EdU-positive cells/Hoechst stained cells) × 100%. Third, a MTT assay was performed. After transfection with miR-192 mimics or negative control, the sheep skeletal muscle SCs were seeded at 1 × 10^3^ in 96-well plate and cultured in GM for 4 days. Every 24 h, 20 μL (5 mg/mL) of MTT solution (Sigma-Aldrich, USA) was added to the cells for 4 h. After discarding the medium, DMSO was added to the cells to dissolve the crystals. The absorbance of each well was measured on a microplate reader.

### RNA oligonucleotides and cell transfection

The siRNA against sheep *RB1*, an siRNA control (a nonspecific duplex), ovine and murine miR-192 mimics (double-stranded RNA oligonucleotides), double-stranded negative control, 2′-O-methyl antisense oligonucleotides against miR-192, and a single-stranded negative control were all purchased from GenePharma (GenePharma, China). Transfection was performed with the Lipofectamine 3000 reagent (Invitrogen, USA) combined with 200 nM of siRNA, 50 nM of miRNA mimics, or 100 nM of 2′-O-methyl antisense oligonucleotides. All the procedures were performed according to the manufacturer’s protocols.

### Dual-luciferase reporter assay

A fragment of 3′-UTR of *RB1* (554 bp) containing the binding site of miR-192 was amplified from the Duolang sheep genomic DNA, which was extracted from *longissimus dorsi* muscle and cloned into the psicheck-2 plasmid (Promega, USA) using the XhoI and NotI restriction sites. *RB1* 3′-UTR was amplified using the forward primer 5′-GGCGCTCGAGTTAACTTCAGCATGGTCTT-3′ and the reverse primer 5′-AATGCGGCCGCGAAGTTCCTTAAATTCTGA-3′. The PCR product was cloned into the vector downstream of the Renilla Luciferase ORF. The mutant *RB1* 3′-UTR reporter was obtained by changing the miR-192 binding site from TAGGTCA to ATCCAGT. *RB1* 3′-UTR binding site mutations were introduced using the KOD-plus mutagenesis kit (Toyobo, Japan) according to the manufacturer’s instructions.

In dual-luciferase reporter (psicheck-2 plasmid), the *Renilla* luciferase is used as a reporter gene and the firefly luciferase is used as a reference gene for control. Luciferase reporter experiments were performed in HEK293 cells that were co-transfected with 100 nM of the miR-192 mimics or NC and 100 ng of the wild-type or mutant 3′-UTR luciferase reporter using Lipofectamine 3000 reagent in 96-well plates. After transfection for 48 h, the relative activities of luciferase were quantified using a Dual-Luciferase Assay System (Promega, USA), according to the manufacturer’s protocols.

### Statistical analysis

All data are shown as mean ± standard deviation (SD), and are based on at least three replicates for each treatment. Unpaired or paired two-tailed t-test was used for individual comparisons. One-way analysis of variance (ANOVA) followed by Duncan’s test was used for multiple comparisons.

## Additional Information

**How to cite this article**: Zhao, Q. *et al*. Expression profiling and functional characterization of miR-192 throughout sheep skeletal muscle development. *Sci. Rep.* **6**, 30281; doi: 10.1038/srep30281 (2016).

## Supplementary Material

Supplementary Information

Supplementary Table S1

Supplementary Table S2

Supplementary Table S3

Supplementary Table S4

Supplementary Table S5

Supplementary Table S6

Supplementary Table S7

Supplementary Table S8

Supplementary Table S9

Supplementary Table S10

Supplementary Table S11

Supplementary Table S12

Supplementary Table S13

## Figures and Tables

**Table 1:** The most abundant miRNAs found in sheep skeletal muscle at the following developmental stages: fetus 90 days (F90), fetus 110 days (F110), lamb 40 days (L40), and adult 3 years (A3Y).

miR name	3Y	L40	F110	F90	Total counts
oar-miR-3958-3p	213	14056	746348	1254154	2014771
oar-miR-432	45	1295	329374	302933	633647
oar-miR-127	2174	53927	205964	277836	539901
oar-miR-543-3p	56	401	91290	93672	185419
oar-miR-411a-5p	7765	95122	51080	21456	175423
oar-miR-495-3p	213	3919	48300	70236	122668
oar-miR-379-5p	1456	11146	31878	43496	87976
oar-miR-381-3p	2361	66028	1734	1033	71156
oar-miR-382-3p	20	218	54863	11757	66858
oar-miR-382-5p	53	1126	31455	25020	57654
oar-miR-485-5p	16	209	26619	22042	48886
oar-miR-433-3p	15	124	16088	27507	43734
oar-miR-409-3p	65	384	16414	11270	28133
oar-miR-494-3p	861	8535	7222	8987	25605
oar-miR-493-5p	96	1935	8820	13091	23942
