# Supplementary material for: Expression profiling and functional characterization of miR-192 throughout sheep skeletal muscle development
Source: Sci Rep. 2016 Jul 25;6:30281. doi: 10.1038/srep30281 (PMC4958965; doi:10.1038/srep30281)
Supplement: Supplementary Information [file srep30281-s1.pdf]

Supplementary Information

Expression profiling and functional characterization of miR-192 throughout sheep skeletal muscle development

Qian Zhao[1], Ye Kang[1,], Hong-Yang Wang[1], Wei-Jun Guan[1], Xiang-Chen Li[1], Lin Jiang[1], Xiao-Hong He[1], Ya-Bin Pu[1], Jian-Lin Han[1,2], Yue-Hui Ma[1*], Qian-Jun Zhao[1*]

[1]Institute of Animal Science, Chinese Academy of Agricultural Sciences, Beijing, 00193, China. [2]CAAS-ILRI Joint Laboratory on Livestock and Forage Genetic Resources, Institute of Animal Science, Chinese Academy of Agricultural Sciences, Beijing, 100193, China. Correspondence and requests for materials should be addressed to Y.H.M. (email: yuehui.ma@263.net) or Q.J.Z. (email: zhaoqianjun@caas.cn)

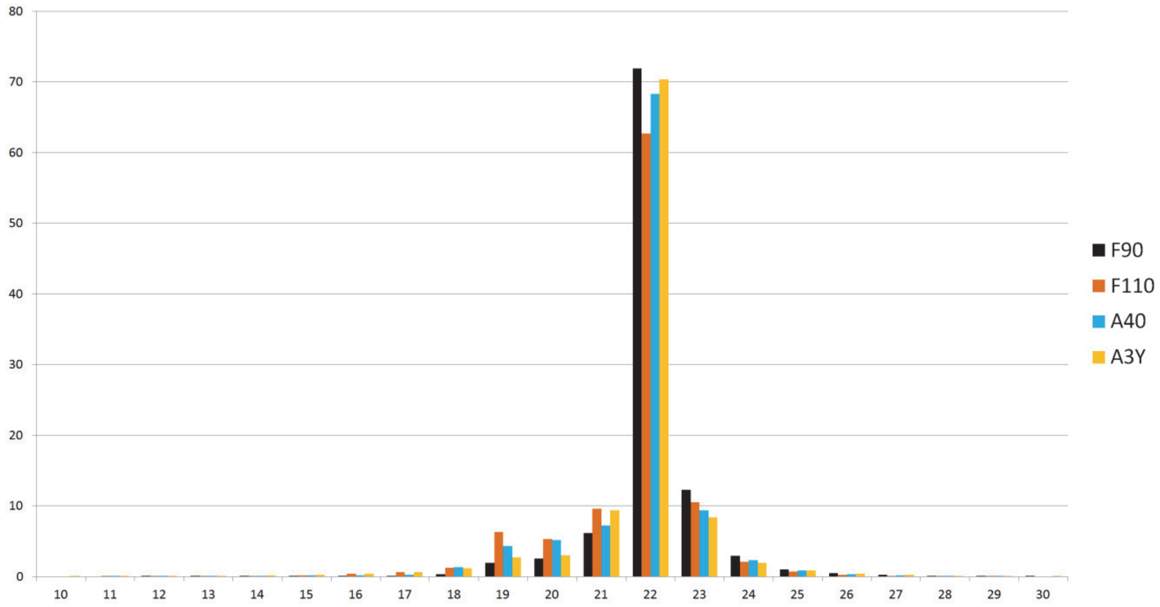

Figure S1. Length distribution of small RNAs. F90: fetus 90 days libraries, F110: fetus 110 days libraries, L40: lamb 40 days libraries and A3Y : adult 3 years libraries.

A

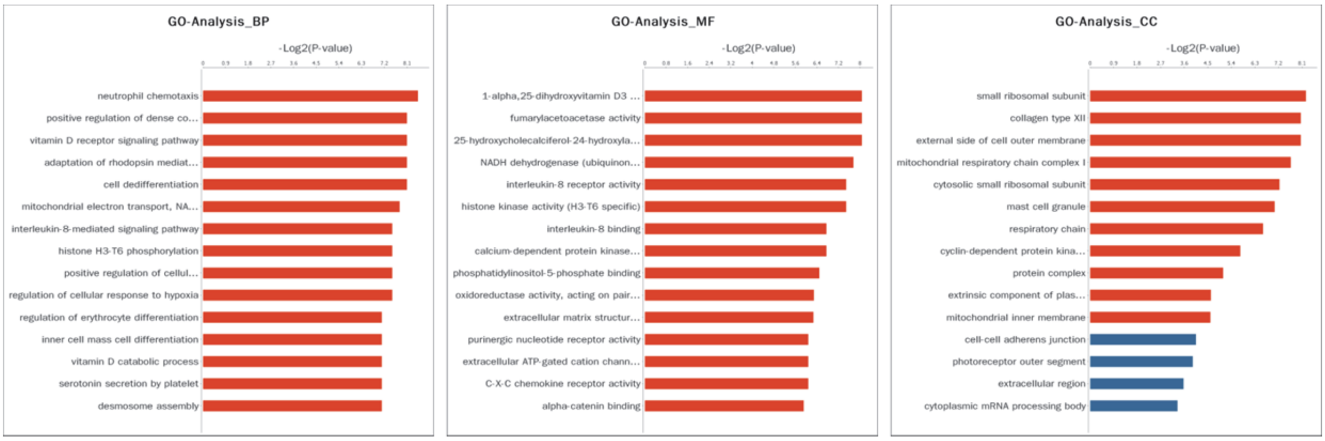

B

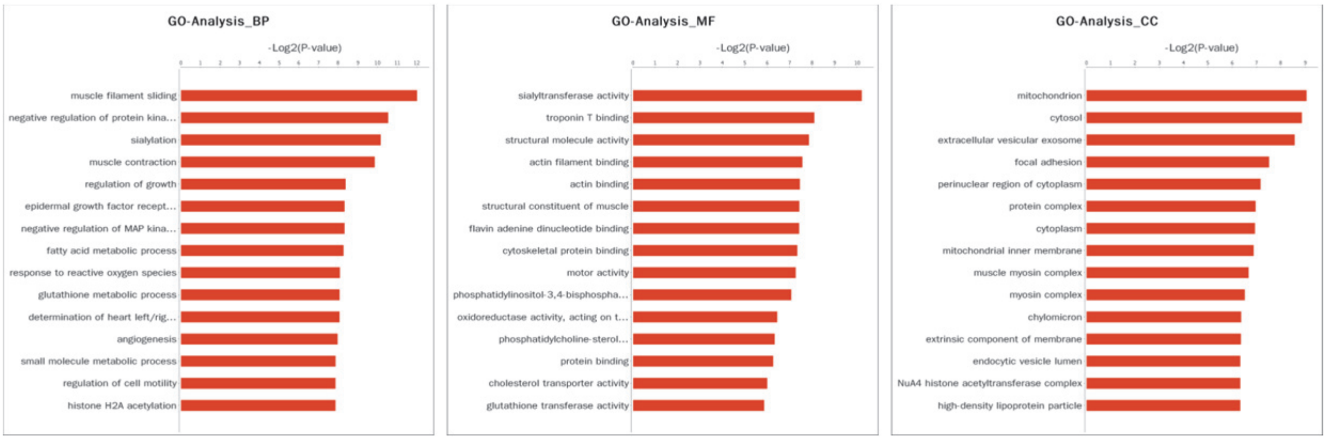
GO-Analysis_BP
-Log2(P-value)
muscle filament sliding
negative regulation of protein kina...
sialylation
muscle contraction
regulation of growth
epidermal growth factor recept...
negative regulation of MAP kina...
fatty acid metabolic process
response to reactive oxygen species
glutathione metabolic process
determination of heart left/rig...
angiogenesis
small molecule metabolic process
regulation of cell motility
histone H2A acetylation
GO-Analysis_MF
-Log2(P-value)
sialyltransferase activity
troponin T binding
structural molecule activity
actin filament binding
actin binding
structural constituent of muscle
flavin adenine dinucleotide binding
cytoskeletal protein binding
motor activity
phosphatidylinositol-3,4-bisphospha...
oxidoreductase activity, acting on t...
phosphatidylcholine-sterol...
protein binding
cholesterol transporter activity
glutathione transferase activity
GO-Analysis_CC
-Log2(P-value)
mitochondrion
cytosol
extracellular vesicular exosome
focal adhesion
perinuclear region of cytoplasm
protein complex
cytoplasm
mitochondrial inner membrane
muscle myosin complex
myosin complex
chylomicron
extrinsic component of membrane
endocytic vesicle lumen
NuA4 histone acetyltransferase complex
high-density lipoprotein particle

C

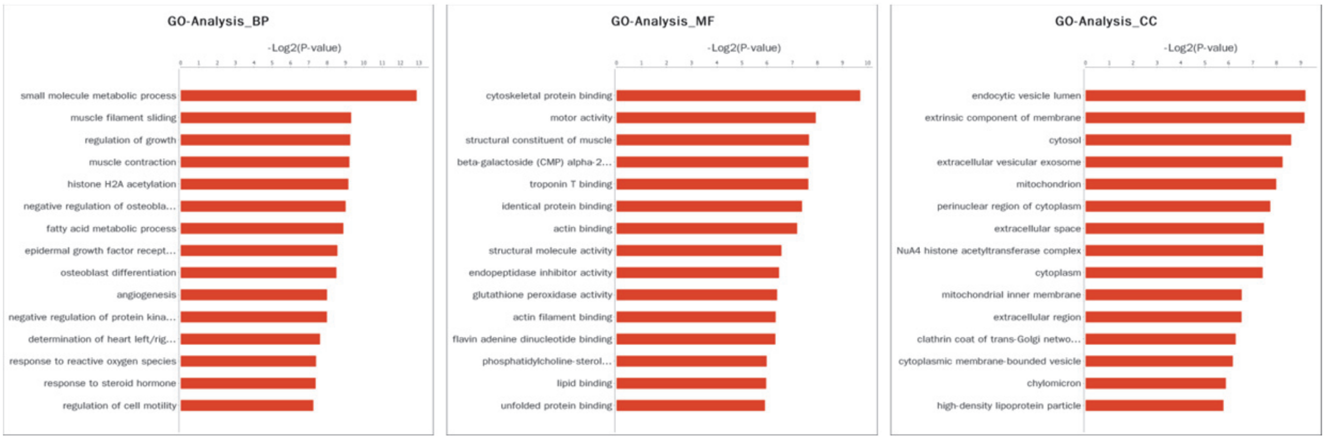
GO-Analysis_BP
-Log2(P-value)
small molecule metabolic process
muscle filament sliding
regulation of growth
muscle contraction
histone H2A acetylation
negative regulation of osteobla...
fatty acid metabolic process
epidermal growth factor recept...
osteoblast differentiation
angiogenesis
negative regulation of protein kina...
determination of heart left/rig...
response to reactive oxygen species
response to steroid hormone
regulation of cell motility
GO-Analysis_MF
-Log2(P-value)
cytoskeletal protein binding
motor activity
structural constituent of muscle
beta-galactoside (CMP) alpha-2...
troponin T binding
identical protein binding
actin binding
structural molecule activity
endopeptidase inhibitor activity
glutathione peroxidase activity
actin filament binding
flavin adenine dinucleotide binding
phosphatidylcholine-sterol...
lipid binding
unfolded protein binding
GO-Analysis_CC
-Log2(P-value)
endocytic vesicle lumen
extrinsic component of membrane
cytosol
extracellular vesicular exosome
mitochondrion
perinuclear region of cytoplasm
extracellular space
NuA4 histone acetyltransferase complex
cytoplasm
mitochondrial inner membrane
extracellular region
clathrin coat of trans-Golgi netwo...
cytoplasmic membrane-bounded vesicle
chylomicron
high-density lipoprotein particle

D

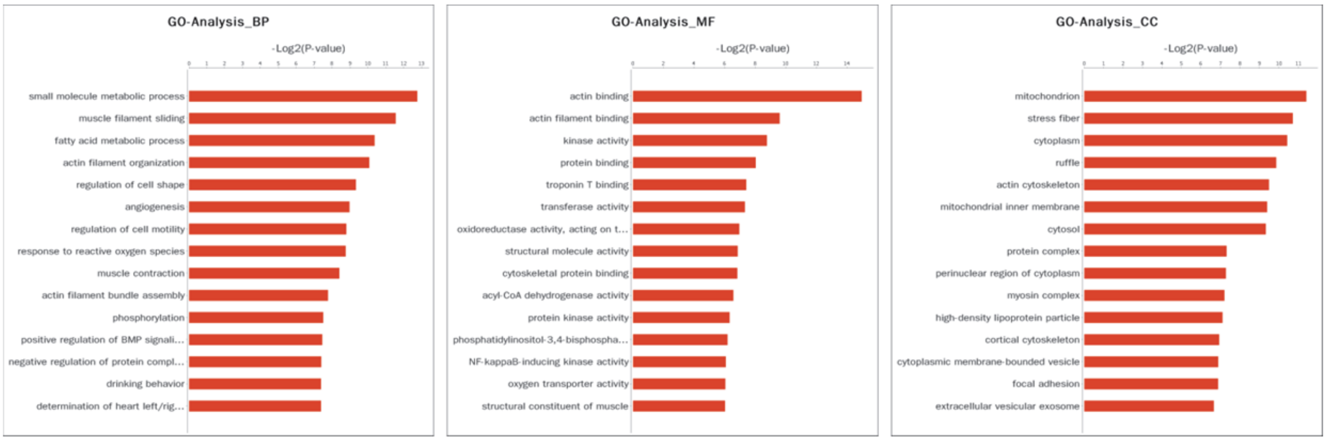
GO-Analysis_BP
-Log2(P-value)
small molecule metabolic process
muscle filament sliding
fatty acid metabolic process
actin filament organization
regulation of cell shape
angiogenesis
regulation of cell motility
response to reactive oxygen species
muscle contraction
actin filament bundle assembly
phosphorylation
positive regulation of BMP signali...
negative regulation of protein compl...
drinking behavior
determination of heart left/rig...
GO-Analysis_MF
-Log2(P-value)
actin binding
actin filament binding
kinase activity
protein binding
troponin T binding
transferase activity
oxidoreductase activity, acting on t...
structural molecule activity
cytoskeletal protein binding
acyl-CoA dehydrogenase activity
protein kinase activity
phosphatidylinositol-3,4-bisphospha...
NF-kappaB-inducing kinase activity
oxygen transporter activity
structural constituent of muscle
GO-Analysis_CC
-Log2(P-value)
mitochondrion
stress fiber
cytoplasm
ruffle
actin cytoskeleton
mitochondrial inner membrane
cytosol
protein complex
perinuclear region of cytoplasm
myosin complex
high-density lipoprotein particle
cortical cytoskeleton
cytoplasmic membrane-bounded vesicle
focal adhesion
extracellular vesicular exosome

E

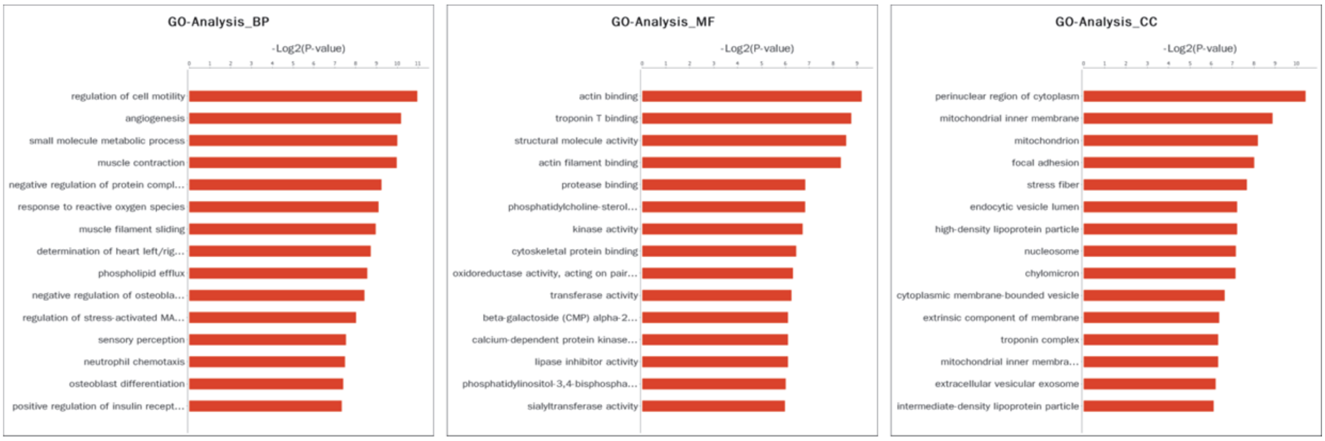
GO-Analysis_BP
-Log2(P-value)
regulation of cell motility
angiogenesis
small molecule metabolic process
muscle contraction
negative regulation of protein compl...
response to reactive oxygen species
muscle filament sliding
determination of heart left/rig...
phospholipid efflux
negative regulation of osteobla...
regulation of stress-activated MA...
sensory perception
neutrophil chemotaxis
osteoblast differentiation
positive regulation of insulin recept...
GO-Analysis_MF
-Log2(P-value)
actin binding
troponin T binding
structural molecule activity
actin filament binding
protease binding
phosphatidylcholine-sterol...
kinase activity
cytoskeletal protein binding
oxidoreductase activity, acting on pair...
transferase activity
beta-galactoside (CMP) alpha-2...
calcium-dependent protein kinase...
lipase inhibitor activity
phosphatidylinositol-3,4-bisphospha...
sialyltransferase activity
GO-Analysis_CC
-Log2(P-value)
perinuclear region of cytoplasm
mitochondrial inner membrane
mitochondrion
focal adhesion
stress fiber
endocytic vesicle lumen
high-density lipoprotein particle
nucleosome
chylomicron
cytoplasmic membrane-bounded vesicle
extrinsic component of membrane
troponin complex
mitochondrial inner membra...
extracellular vesicular exosome
intermediate-density lipoprotein particle

F

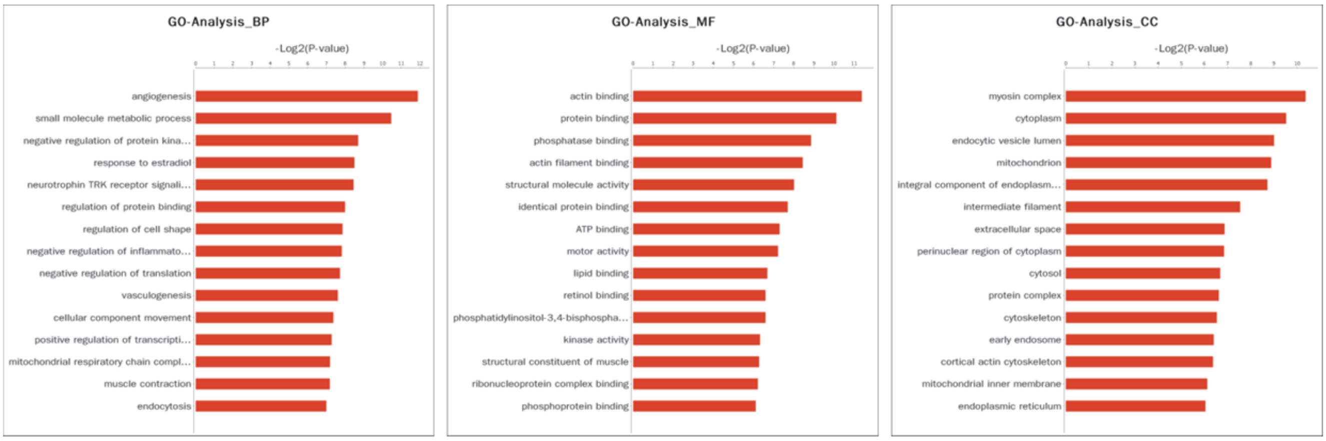

Figure S2. GO functional analysis of the target genes of SDEmiRs. GO biological process (BP), molecular function (MF) and cellular component (CC) were shown. (A), (B), (C), (D), (E) and (F) represent GO item for target genes of SDEmiRs between F90 and F110, F90 and A40, F90 and A3Y, F110 and A40, F110 and A3Y, and A40 and A3Y, respectively. Red column refers to the significantly enriched pathway with $p < 0.05$ while blue refers to the insignificantly enriched ones with $p \geq 0.05$.

A

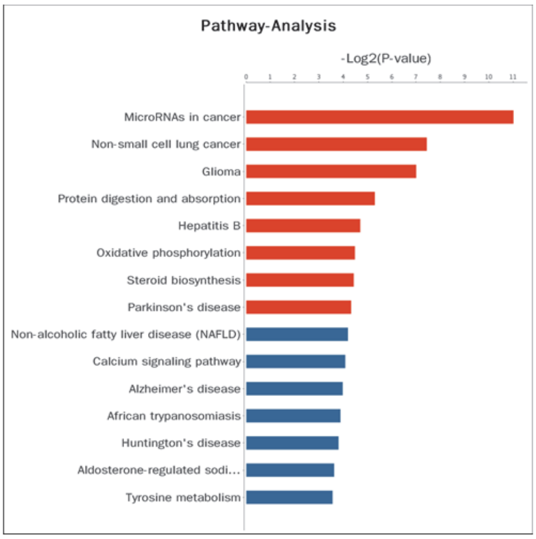

B

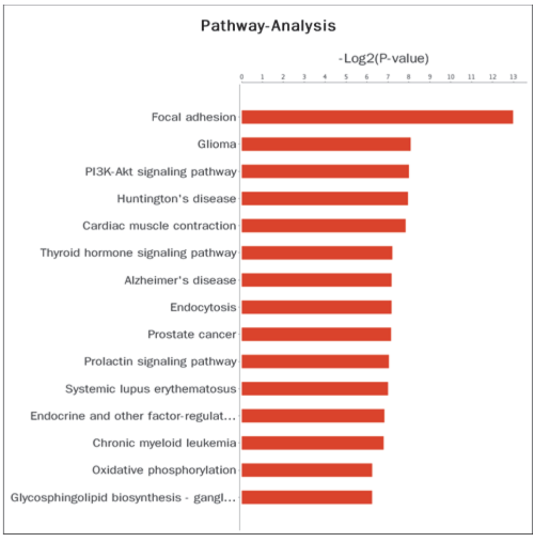

C

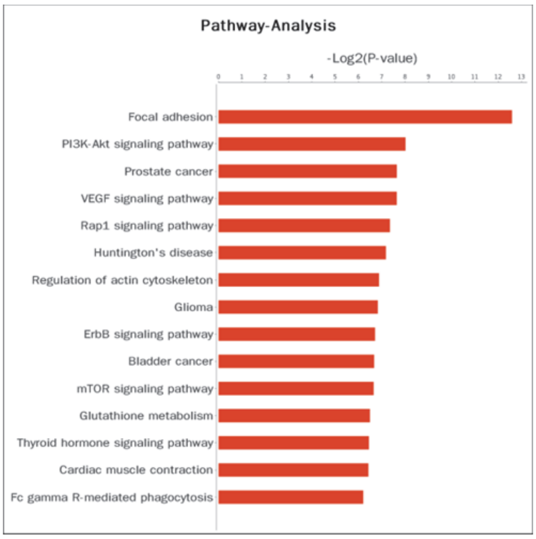

D

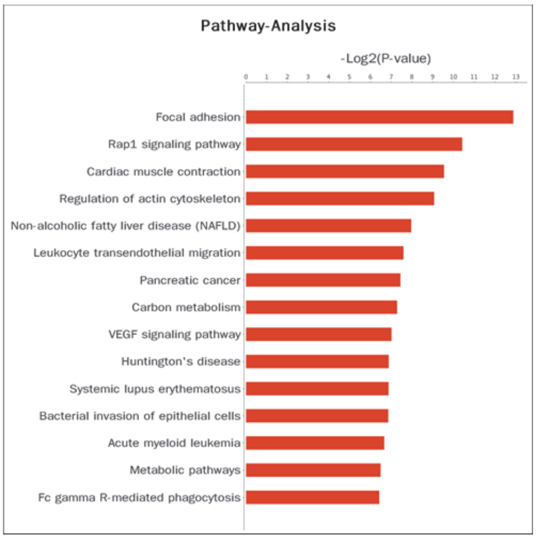

E

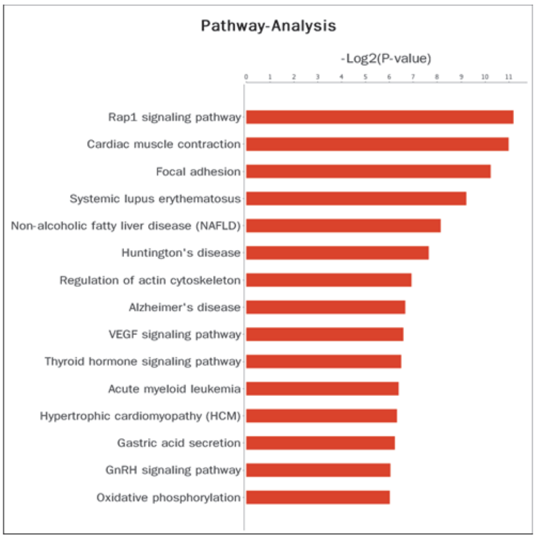

F

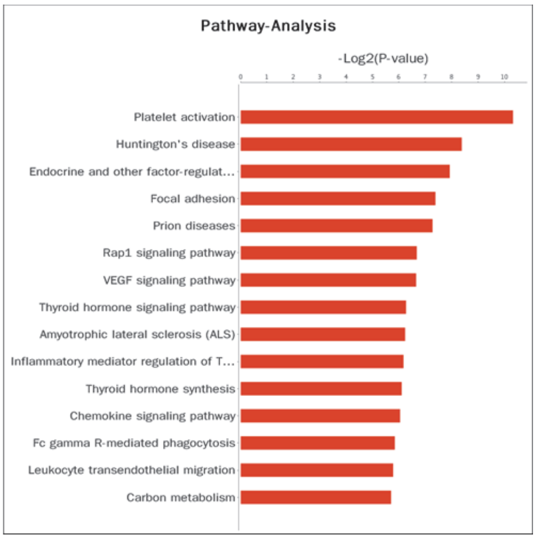

Figure S3. KEGG pathway analysis of the target genes of SDEmiRs. (A), (B), (C), (D), (E) and (F) represent KEGG pathway analysis for the target genes of SDEmiRs between F90 and F110, F90 and A40, F90 and A3Y, F110 and A40, F110 and A3Y, and A40 and A3Y, respectively. Red column refers to the significantly enriched pathway with $p < 0.05$ while blue refers to the insignificantly enriched ones with $p \geq 0.05$.

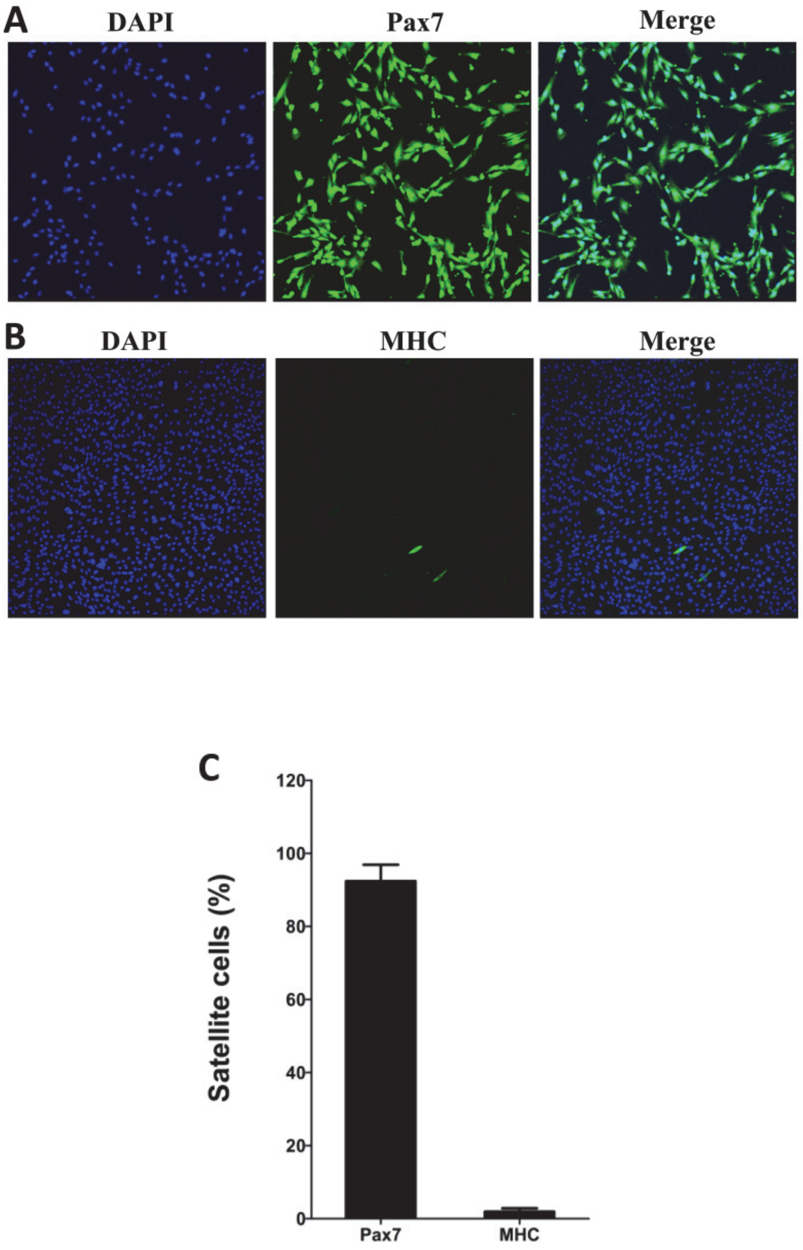

Figure S4. Identification and characterization of skeletal muscle satellite cells (SCs). (A) The isolated SCs were immunostained for Pax7 (green) and DAPI (blue). (B) The isolated SCs were immunostained for MHC (green) and DAPI (blue). (C) Quantification of the distribution of isolated SCs population that express Pax7 or MHC, respectively. The results are shown as the mean ± SD. of three replicates.

miR-192

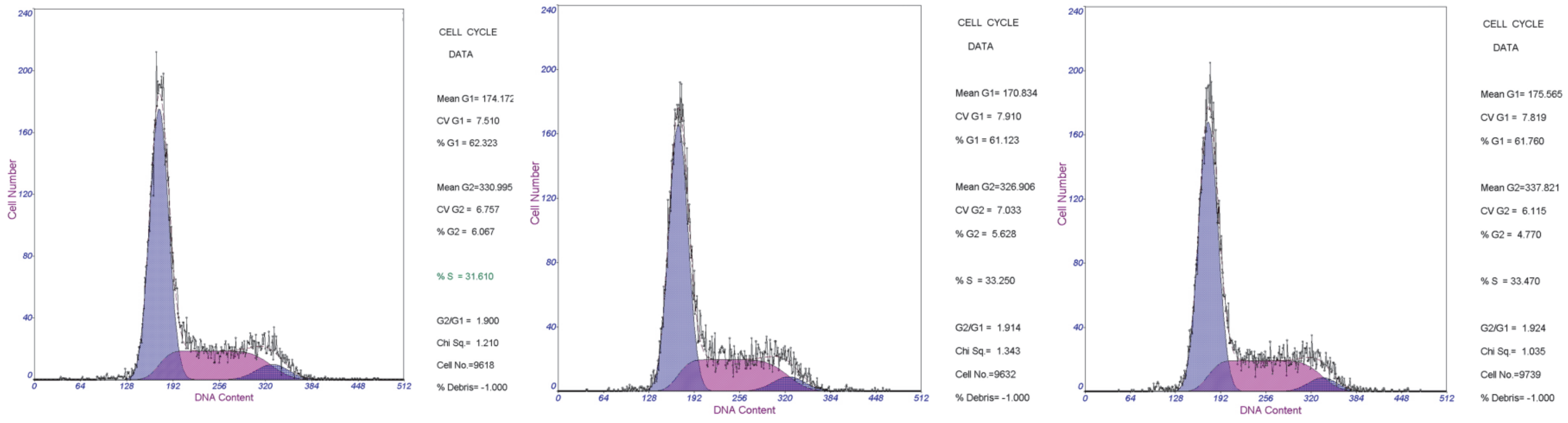

NC

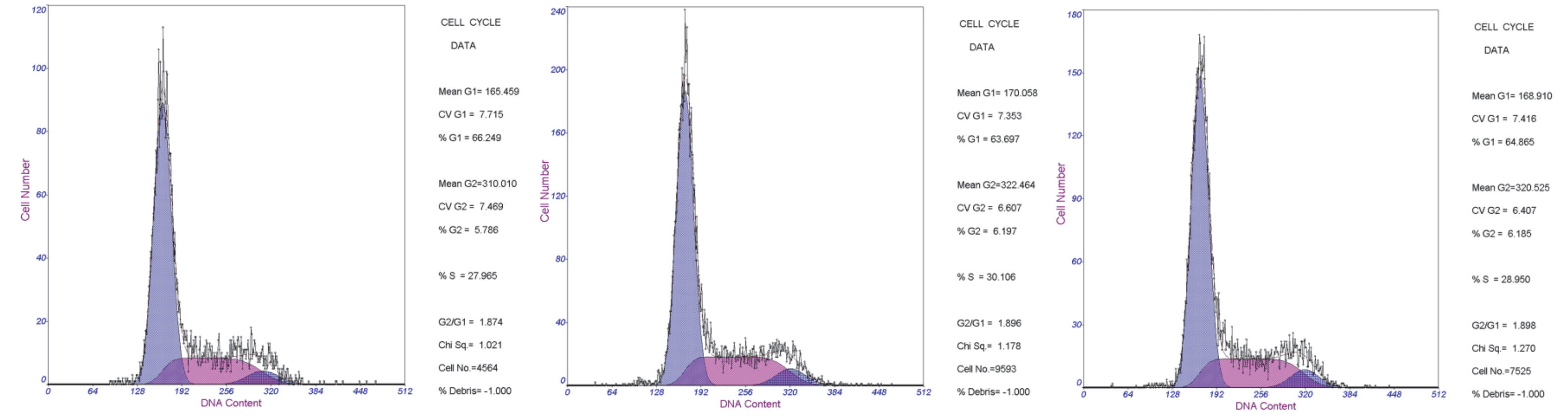

Figure S5. The histograms of cell cycle were analyzed by flow cytometry. SCs were harvested for cell cycle analysis after the cells were transfected at GM and continuously cultured for 48.

Table S14 – Primers of genes for qPCR

Gene name	mRNA accession number	Sequence (5'→3')	Amplicon length (bp)
MHC	AB058897	F: 5'-CTTCGTGGCGGACCCTAAG-3'	101
		R: 5'-CAGTTACTGTCGCCCCAGCT-3'	
Myogenin	GU550517.1	F: 5'- AATGAAGCCTTCGAGGCCC -3'	101
		R: 5'- CGCTCTATGTACTGGATGGCG -3'	
RB1	EU525169.1	F: 5'- AAGGACCGAGAAGGACCAGT -3'	140
		R: 5'- TTTGCACGTGGAGTTGACG -3'	
ACTB	NM_001009784	F: 5'- TGGGCATGGAATCCTG-3'	194
		R: 5'- GGCGCGATGATCTTGAT-3'	
GAPDH	NM_001190390	F:5'-CAAGTTCCACGGCACAGTCA-3'	249
		R: 5'-TGGTTCACGCCCATCACAA-3'	

Table S15 – Primers of miRNAs for qPCR

miRNA name	Sequence (5'→3')
miR-127 (sheep)	RT:5'-CTCAACTGGTGTCGTGGAGTCGGCAATTCAGTTGAGAGCCAAGC-3'
	F: 5'-CCTAGATCGGATCCGTCTGA-3'
	R: 5'-TGGTGTCGTGGAGTCGGCAAT-3'
miR-495-3p (sheep)	RT:5'-CTCAACTGGTGTCGTGGAGTCGGCAATTCAGTTGAGAAGAAGTG-3'
	F: 5'-CGGCGGGAAACAAACATGGTG-3'
	R: 5'-TGGTGTCGTGGAGTCGGCAAT-3'
miR-503 (sheep)	RT: 5'-CTCAACTGGTGTCGTGGAGTCGGCAATTCAGTTGAGGCAGTACT-3'
	F: 5'-TTAGCGTGTAGCAGCGGGAAC-3'
	R: 5'-TGGTGTCGTGGAGTCGGCAAT-3'
miR-3958-3p (sheep)	RT:5'-TCAACTGGTGTCGTGGAGTCGGCAATTCAGTTGAGAGAGATCA-3'
	F: 5'- CGGCTCGAAGATATTGCACGGT-3'
	R: 5'-TGGTGTCGTGGAGTCGGCAAT-3'
miR-433-3p (sheep)	RT: 5'- TCAACTGGTGTCGTGGAGTCGGCAATTCAGTTGAGACACCGAG-3'
	F: 5'- CGGTTCGATCATGATGGGCTC-3'
	R: 5'-TGGTGTCGTGGAGTCGGCAAT-3'
miR-382-5p (sheep)	RT: 5'-CTCAACTGGTGTCGTGGAGTCGGCAATTCAGTTGAGCGAATCCA-3'
	F: 5'- GCGGACTGAAGTTGTTCGTGG-3'
	R: 5'-TGGTGTCGTGGAGTCGGCAAT-3'
miR-299-3p (sheep)	RT: 5'-CTCAACTGGTGTCGTGGAGTCGGCAATTCAGTTGAGAAGCGGTT-3'
	F: 5'- CGCTGGTATGTTGGGACGGTA-3'
	R: 5'-TGGTGTCGTGGAGTCGGCAAT-3'
miR-125b (sheep)	RT: 5'-CTCAACTGGTGTCGTGGAGTCGGCAATTCAGTTGAGAAGTGGAT-3'
	RT: 5'-CTCAACTGGTGTCGTGGAGTCGGCAATTCAGTTGAGAAGTGGAT-3'
	R: 5'-TGGTGTCGTGGAGTCGGCAAT-3'
miR-1 (sheep)	RT: 5'-CTCAACTGGTGTCGTGGAGTCGGCAATTCAGTTGAGTACACTTT-3'
	F: 5'- GCGGGCAGCGTGGAATGTAAA-3'
	R: 5'-TGGTGTCGTGGAGTCGGCAAT-3'
miR-206 (sheep)	RT:5'-CTCAACTGGTGTCGTGGAGTCGGCAATTCAGTTGAGCCACACAC-3'
	F: 5'-GCGGCTGGTGGAATGTAAGGAA-3'
	F: 5'-GCGGCTGGTGGAATGTAAGGAA-3'
miR-192 (sheep)	RT:5'-CTCAACTGGTGTCGTGGAGTCGGCAATTCAGTTGAGGCTGTCAA-3'
	F: 5'- GTGGCGGGCTGACCTATGAAT-3'
	R: 5'- TGGTGTCGTGGAGTCGGCAAT-3'
miR-192 (mouse)	RT:5'-CTCAACTGGTGTCGTGGAGTCGGCAATTCAGTTGAGGGCTGTCA-3'
	F: 5'- GTGGCGGGCTGACCTATGAAT-3'
	R: 5'-TGGTGTCGTGGAGTCGGCAAT-3'
